# Adapting a simple surgical manual tool to a 3D printed implantology protocol: the use of a universal screwdriver for fixation of custom-made laser sintered titanium subperiosteal implants

**DOI:** 10.1186/s41205-022-00159-3

**Published:** 2022-10-27

**Authors:** Mustafa Ayna, Aydin Gülses

**Affiliations:** 1 Dr. Ayna Implantology Clinic, Duisburg, Germany; 2 Department of Oral and Maxillofacial Surgery, Universitätsklinikum Schleswig Holstein, Christian Albrechts University, Campus Kiel, Arnold-Heller-Straße 3, 24105 Kiel, Germany

## Abstract

**Purpose:**

Current paper aims to describe a simple technique used for the fixation of the screws of a customized implant via a universal screw driver (BoneTrust® Easy Screw according to Dr. Bayer, Medical Instinct®, GmbH, Germany) to simplify the surgical placement of the customized implants.

**Methods:**

The insertion of the drilling screws for the retention of the implant with angulated handpiece into the palatinal region or zygomatic buttress were performed with universal screw driver.

**Results:**

The retention screws could be inserted with a proper angulation without interfering with the surrounding tissues. The technique described herein has significantly simplified the surgical intervention.

**Conclusion:**

The insertion of the drilling screws for the retention of the implant with angulated handpiece into the palatinal region or zygomatic buttress could be challenging, thus the anatomical structures and the insufficient length of the handpiece could interfere with the placement of the screw with a proper angulation. This problem could be easily managed with the use of universal screw driver.

## Introduction

Atrophy of the edentulous jaws, which mostly necessitates a vertical and/or horizontal augmentation of the alveolar ridge to allow a dental implant rehabilitation, present one of the main challenges in the daily dental practice. Moreover, the diversity of these defects urges the clinician to seek and precisely perform patient-specific-solutions to reclaim an anatomically appropriate implant recipient site.

Despite the fact that novel guided bone regeneration techniques via the use of different volume-stable bone substitutes and/or stabilizing systems such as titanium-reinforced ePTFE membranes could offer many clinical solutions, the reconstruction of a completely edentulous alveolar ridge or excessive defects of the jaws as a result of ablative surgeries or trauma are not manageable without using particulate or block autogenous bone grafts. However, due to the possible anatomical and morphological complexity of the jaws, European Association of Dental implantologists developed a therapy-centered defect classification (The Cologne Classification of Alveolar Ridge Defects-CCARD) and a chart of recommendation of different therapy techniques for the respective defect class [1]. Briefly, CCARD uses three- part codes to describe the effect of the alveolar ridge as comprehensively as possible with a view to existing therapeutic options and describes each defect by a single defect code consisting of letters and numbers:

Part 1: Orientation of the defect

- h: horizontal
- V: vertical
- C: combined
- S (or + S): sinus area

Part 2: Reconstruction needs associated with the defect

- 1. low: < 4 mm
- 2. medium: 4–8 mm

- 3. high: > 8 mm)

Part 3: Relation of augmentation and defect region

- i: internal, inside the contour

- e: external, outside the ridge contour

According to this classification, the bony reconstruction of the vertical and combined defects inside the maxillomandibular ridge contour greater than 4 mm and defects outside the maxillomandibular ridge contour require the use of bone blocks or the performance of a distraction osteogenesis. However, it is very well known that obtaining an autologous bone graft requires a second surgical intervention and is still associated with the risk donor site morbidity. Additionally, the duration of the treatment is another fact to discuss thus an implant insertion could be performed at least 4 to 6 months after augmentation with autologous bone grafts [2]. Despite comprehensive surgical and prosthetic guidelines and developments in materials sciences [3], there is an ongoing need for minimal invasive, timesaving, and case-specific solutions in the dental management of patients with bony defects of the jaws.

The idea of prosthetic rehabilitation of edentulous jaws by using subperiostally placed implants is not a novel treatment modality [4]. However, within the last decade, thanks to the advancements in the 3D printing and dental digital workflow technologies, prefabrication of customized subperiostal implants has gained their popularity in the management of patients with severe atrophied jaws. It has been estimated that more than 450 patients received a prosthetic rehabilitation with the use of customized subperiostal implants produced by selective laser melting until now [5].

**Fig. 1:**
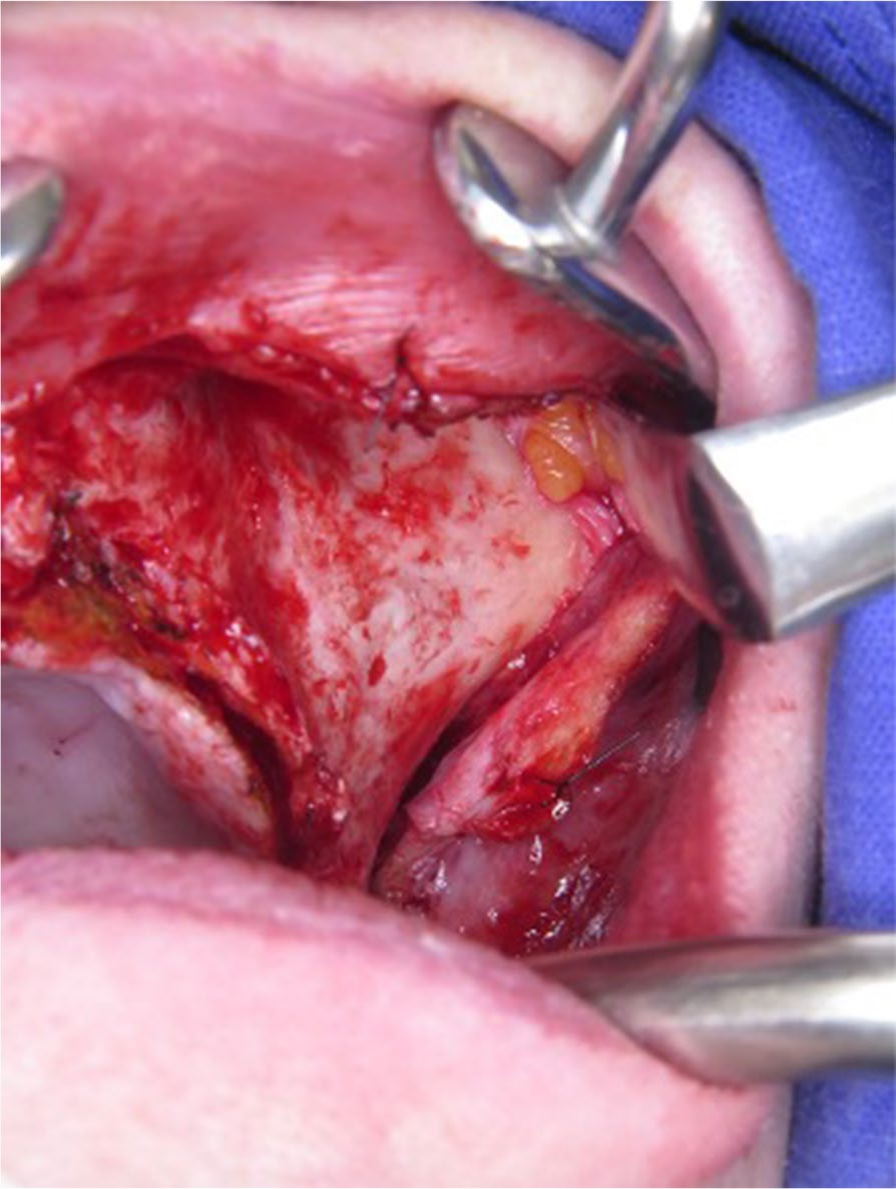
The placement of the over-sized implant requires an overly extended intraoral incision

Despite their advantages such as [5].;

• A perfect adaptation of the implant surface to the load bearing segments of the jaws which also enables immediate functional loading
• Avoiding the need for a second surgical intervention both in guided tissue regeneration and autologous bone augmentation procedures
• Obviating the donor site morbidity secondary to autologous bone graft harvesting,

biological complications such as recurrent infections and mechanical problems such as fractures of both implant and prosthetic components were reported [6]. Additionally, the surgical procedure requires advanced surgical skills due to the difficulties regarding the placement of the over-sized mono-block implant onto the recipient site or insertion of the fixation screws into the ascending zygomatic buttress (Fig. 1) through an overly extended intraoral incision.

Current paper aims to describe a simple technique used for the fixation of the screws of a customized implant via a universal screw driver (BoneTrust^®^ Easy Screw according to Dr. Bayer, Medical Instinct^®^, GmbH, Germany) to simplify the surgical placement of the customized implants.

## Technical note

**Fig. 2:**
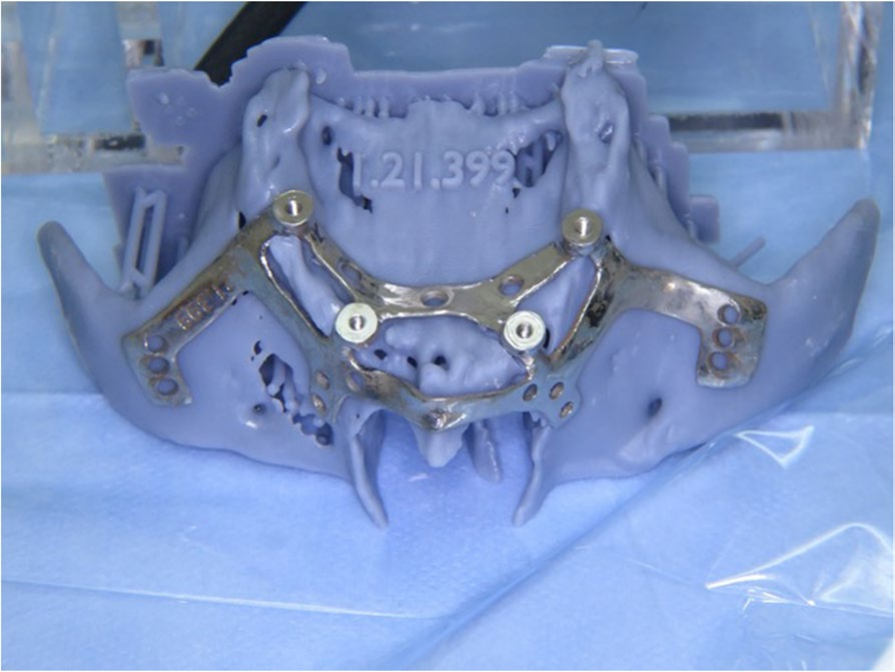
View of the custom-made grade V titanium implant (BoneEasy.®, Ovar, Portugal) manufactured by laser sintering (SLM)

**Fig. 3:**
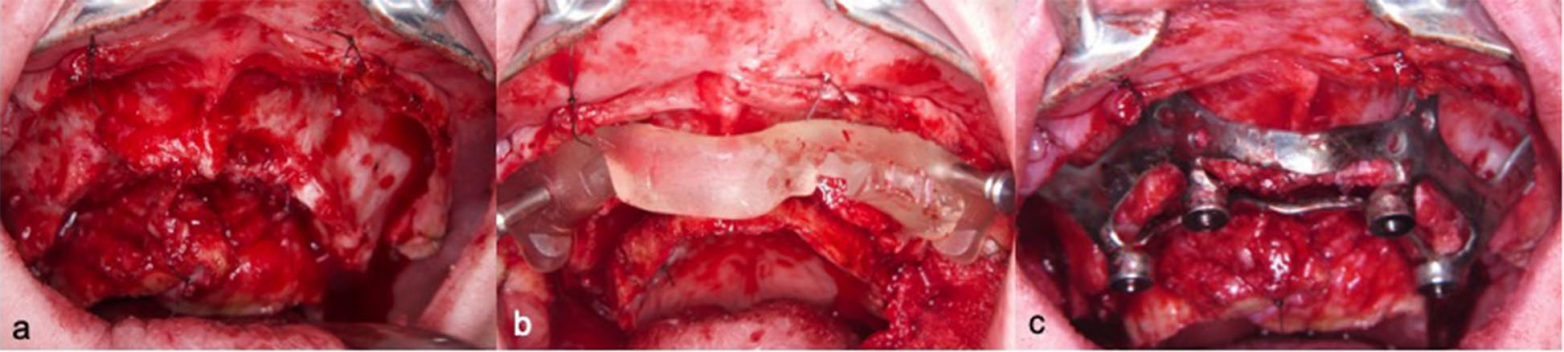
**a**. An extended full-thickness flap was raised to make the bone tissue clearly visible where the custom-made implant was to be placed. **B**. To obtain a perfect adaptation and eliminate the undercuts, a surgical ostectomy guide was used. **c**. The implant was removed from its sterile packaging and placed on the residual bone

**Fig. 4:**
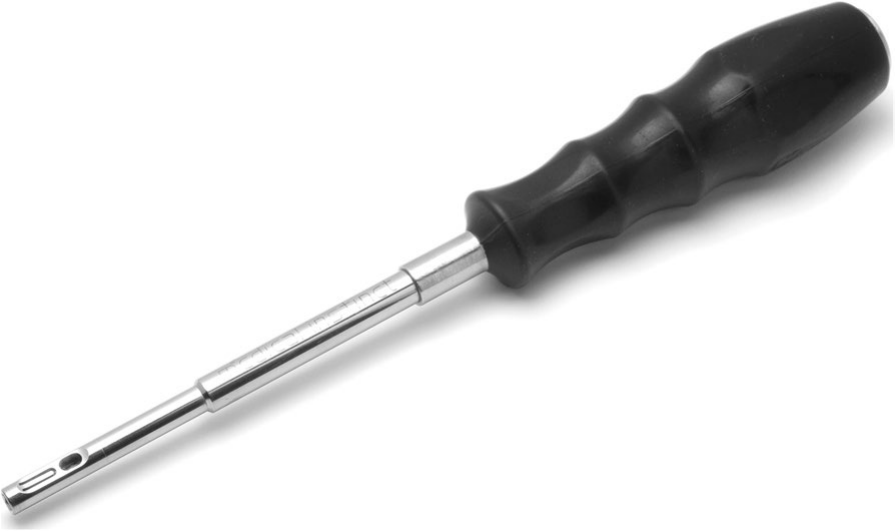
BoneTrust® Easy Screw

**Fig. 5:**
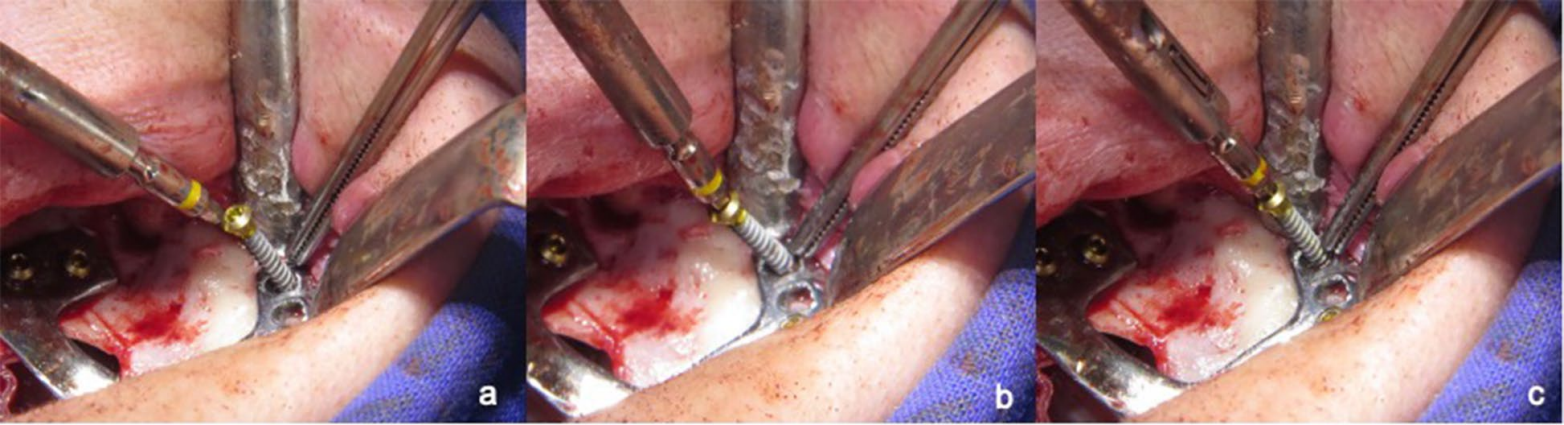
**a**-**c**. Adaptation of the screw driver head engaging the screw

**Fig. 6:**
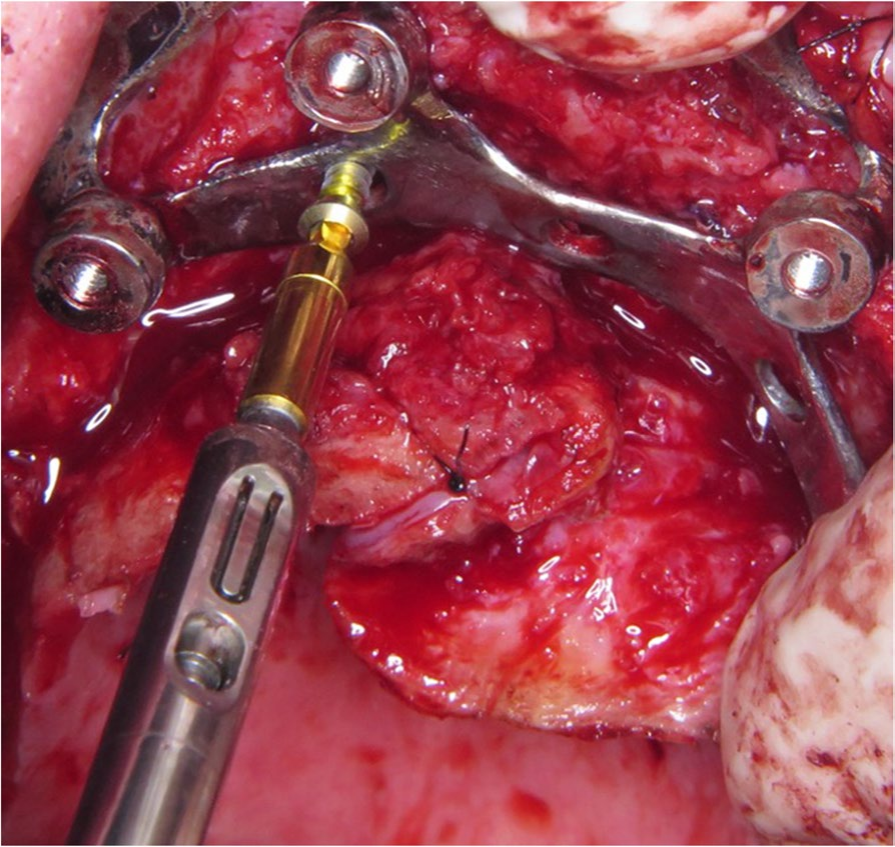
Fixation of the implant with insertion of mini screws, which could be easily placed with “BoneTrust® Easy Screw” thanks to its ability of axis-optimization

**Fig. 7:**
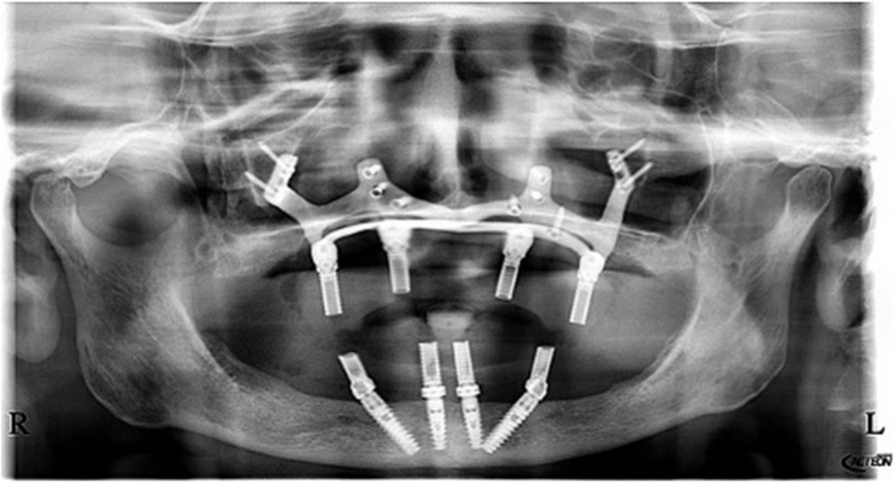
The postoperative panoramic view showed the proper placement of the screws and the implant

**Fig. 8:**
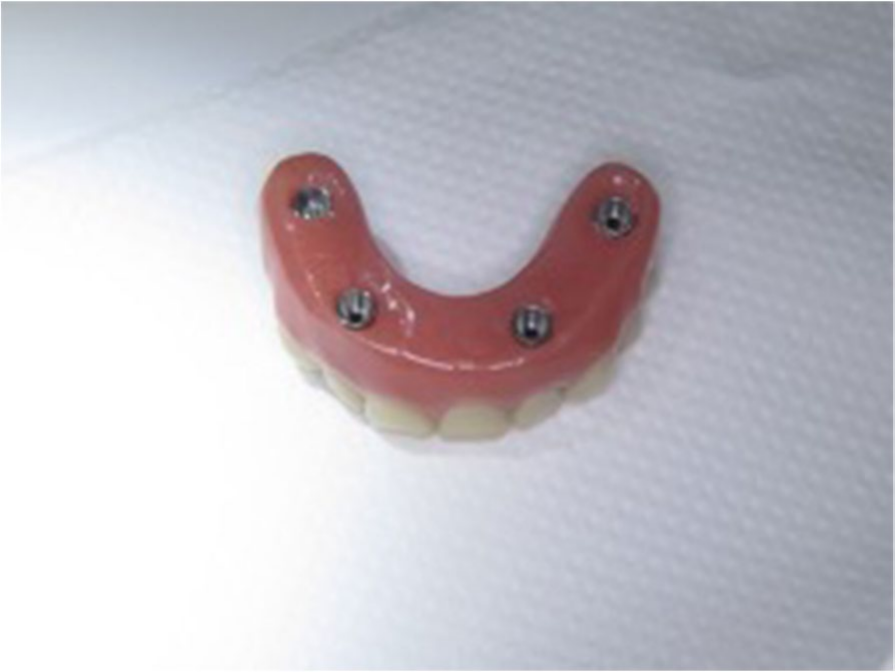
The provisional acrylic prosthesis

**Fig. 9:**
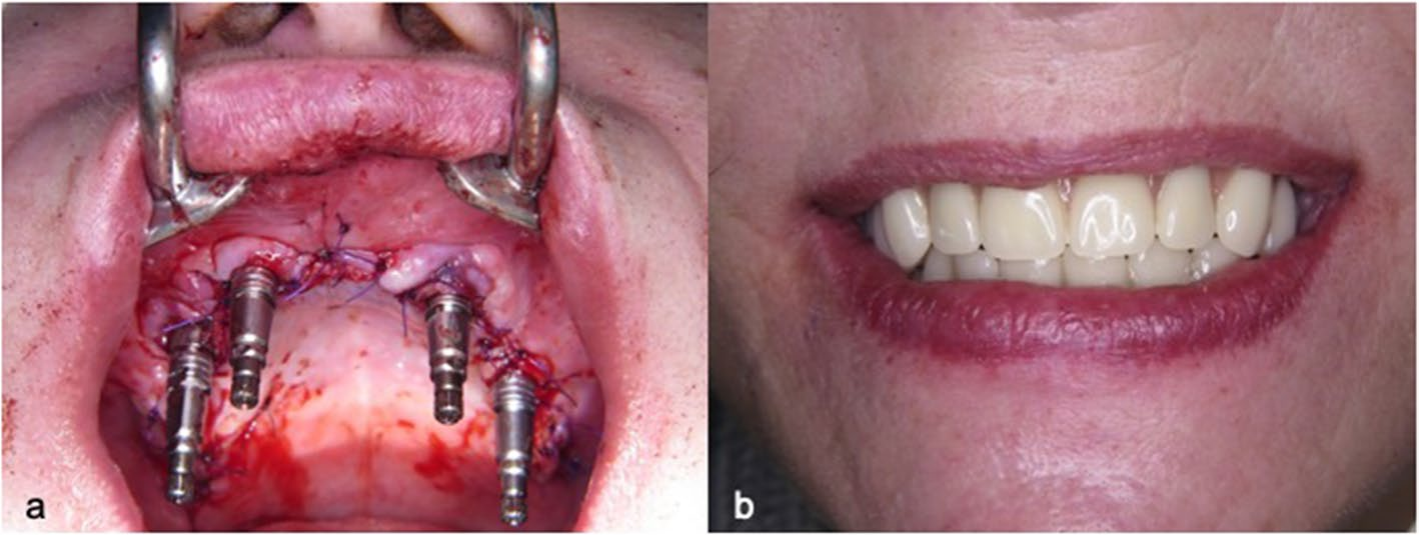
**a** and **b**. The prothesis was mounted after 6 h on the multi-unit abutments

A custom-made grade V titanium implant (BoneEasy^®^, Ovar, Portugal) has been manufactured by laser sintering (SLM/ Selective Laser Melting) technology according to the DICOM files from computed tomography by using computer aided design and segmentation software (Fig. 2). The surgery was performed under local anesthesia and midazolam sedation. A crestal incision was made in the edentulous extremely atrophied maxilla, connected by two deep releasing incisions, mesially and distally. Subsequently, an extended full-thickness flap was raised to make the bone clearly visible where the custom-made implant was to be placed. (Fig. 3a) To obtain a perfect adaptation of the implant and eliminate the undercuts, a surgical ostectomy guide was used (Fig. 3b) The implant was removed from its sterile packaging and placed on the residual bone (Fig. 3c). After the position was checked, the implant has been fixed onto the predetermined area with insertion of mini screws, which could be easily placed with BoneTrust® Easy Screw (Medical Instinct®, Deutschland, GmbH) (Figs. 4, 5 and 6) The postoperative panoramic view showed the proper placement of the screws and the implant (Fig. 7). A provisional acrylic prosthesis (Fig. 8) were mounted in 6 h postoperatively on the multi-unit abutments (Fig. 9 a and b).

## Discussion

According to the manufacturer’s guideline, the ergonomically designed and universally applicable BoneTrust^®^ Easy Screw facilitates the manual screwing in of implants in the anterior area of the jaws thanks to its axis-optimized positioning. Especially in the maxilla, whereas a bone type of D3 or D4 exists, all common instruments from the pilot drill to the carrier required for the implant insertion can be mounted on the screw driver which enables a complete manual work-flow. This protocol might be more comfortable for the patient, thus the need for cooling via an external irrigation could be abandoned. In addition, the screw driver could allow to collect the bone chips accumulated on the drill for a possible augmentation.

A severe bone atrophy which necessitates an autologous bone augmentation for implant insertion can be difficult in elderly or in patients with compromised general health status. Moreover, a further surgical intervention for re-augmentation could be rejected by the patient after a failure of an augmentation. In these cases, subperiosteal implants may be an alternative, due to the digital revolution and the advent of modern digital technologies in dentistry [5, 7, 8]. 3D printing and in particular direct metal laser sintering (DMLS) allows fabrication of custom-made meshes and even implants perfectly adapted to the patient’s specific anatomy. However, the insertion of the drilling screws for the retention of the implant with angulated handpiece into the palatinal region or zygomatic buttress could be challenging, thus the anatomical structures and the insufficient length of the handpiece could interfere with the placement of the screw with a proper angulation. This problem could be easily managed with the use of universal screw driver due to its ability of axis-optimization.

## Conclusion

The universal tool “BoneTrust^®^ Easy Screw” which is compatible with almost all implant systems and could be used from thread cutting through implant insertion to screwing of prosthetic components is yet entrusted with the fixation of customized novel subperiostal implants.

## Data Availability

All visual data is available in the Clinic of Implantology Dr. Mustafa Ayna, Duisburg/ Germany).

## Abbreviations

DICOM: Digital Imaging and Communications in Medicine
DMSL: Direct metal laser sintering
SLM: Selective Laser Melting

## References

[1] Neugebauer J, Nickenig HJ, Zöller JE. Bundesverband der implantologisch tätigen Zahnärzte in Europa (European Association of Dental Implantologists) Praxisleitfaden: Umgang mit Komplikationen bei der implantologischen Behandlung 14. Europäische Konsensuskonferenz (EuCC). 2019;03:1–7.

[2] Kim YK, Ku JK (2020). Ridge augmentation in implant dentistry. J Korean Assoc Oral Maxillofac Surg.

[3] Emmert M, Spille J, Behrens E, Ayna M, Karayürek F, Wiltfang J, Acil Y, Gülses A (2022). Comparative assessment of the primary stability of Straumann BLX implant design using an in vitro sinus lift simultaneous implant insertion model. J Oral Implantol.

[4] Silvestri KD, Carlotti AE (1995). Subperiosteal implant: serving the dental profession for over 50 years. R I Dent J..

[5] Mangano C, Bianchi A, Mangano FG, Dana J, Colombo M, Solop I, Admakin O (2020). Custom-made 3D printed subperiosteal titanium implants for the prosthetic restoration of the atrophic posterior mandible of elderly patients: a case series. 3D Print Med.

[6] Cerea M, Dolcini GA (2018). Custom-made direct metal laser sintering titanium subperiosteal implants: a retrospective clinical study on 70 patients. Biomed Res Int.

[7] Perveen A, Molardi C, Fornaini C (2018). Applications of laser welding in dentistry: a state-of-the-art review. Micromachines (Basel).

[8] Nazarian A (2014). Placement of a modifed subperiosteal implant: a clinical solution to help those with no bone. Dent Today.

